# Dexamethasone exacerbates cerebral edema and brain injury following lithium-pilocarpine induced status epilepticus^☆^

**DOI:** 10.1016/j.nbd.2013.12.001

**Published:** 2014-03

**Authors:** B.A. Duffy, K.P. Chun, D. Ma, M.F. Lythgoe, R.C. Scott

**Affiliations:** a Centre for Advanced Biomedical Imaging (CABI), Department of Medicine, University College London (UCL), UK; b School of Environment and Sustainability, University of Saskatchewan, Canada; c Centre for Medical Image Computing (CMIC), University College London (UCL), UK; d Department of Neurological Sciences, College of Medicine, University of Vermont, Burlington 05405, VT, USA; e UCL Institute of Child Health, University College London, London, UK

**Keywords:** BBB, blood–brain barrier, CSE, convulsive status epilepticus, SE, status epilepticus, NSAIDs, non-steroidal anti-inflammatory drugs, COX-2, cyclooxygenase-2, MRI, magnetic resonance imaging, DEX, dexamethasone, IκB, inhibitor of kappa-B, T_2_, transverse magnetisation relaxation time constant, rHCV, relative hippocampal volume, fse, fast spin-echo, TR, repetition time, FOV, field of view, TE, echo time, TEeff, effective echo time, etl, echo-train length, ROIs, regions of interest, Corticosteroids, Epilepsy, Biomarker, T_2_, MRI, Inflammation

## Abstract

Anti-inflammatory therapies are the current most plausible drug candidates for anti-epileptogenesis and neuroprotection following prolonged seizures. Given that vasogenic edema is widely considered to be detrimental for outcome following status epilepticus, the anti-inflammatory agent dexamethasone is sometimes used in clinic for alleviating cerebral edema. In this study we perform longitudinal magnetic resonance imaging in order to assess the contribution of dexamethasone on cerebral edema and subsequent neuroprotection following status epilepticus. Lithium-pilocarpine was used to induce status epilepticus in rats. Following status epilepticus, rats were either post-treated with saline or with dexamethasone sodium phosphate (10 mg/kg or 2 mg/kg). Brain edema was assessed by means of magnetic resonance imaging (T_2_ relaxometry) and hippocampal volumetry was used as a marker of neuronal injury. T_2_ relaxometry was performed prior to, 48 h and 96 h following status epilepticus. Volume measurements were performed between 18 and 21 days after status epilepticus. Unexpectedly, cerebral edema was worse in rats that were treated with dexamethasone compared to controls. Furthermore, dexamethasone treated rats had lower hippocampal volumes compared to controls 3 weeks after the initial insult. The T_2_ measurements at 2 days and 4 days in the hippocampus correlated with hippocampal volumes at 3 weeks. Finally, the mortality rate in the first week following status epilepticus increased from 14% in untreated rats to 33% and 46% in rats treated with 2 mg/kg and 10 mg/kg dexamethasone respectively. These findings suggest that dexamethasone can exacerbate the acute cerebral edema and brain injury associated with status epilepticus.

## Highlights

• Dexamethasone given following seizures caused a worsening of edema and brain injury.
• This finding also applied to doses dexamethasone as low as 2 mg/kg.
• We show the use of automated hippocampal volume measurement for therapy monitoring.

## Introduction

Inflammation has been suggested to play a major role in epileptogenesis (Ravizza et al., 2011, Vezzani, 2013). Mechanisms for this are thought to occur via leakage of blood serum components into the parenchyma across an impaired blood–brain barrier (BBB) leading to impaired astrocyte function and altered potassium homeostasis (Cacheaux et al., 2009, David et al., 2009, Friedman et al., 2009, Ivens et al., 2007, Seiffert et al., 2004, van Vliet et al., 2007). This leads to the hypothesis that anti-inflammatory therapies which help to alleviate vasogenic edema are likely to be anti-epileptogenic or neuroprotective following convulsive status epilepticus (CSE).

A variety of anti-inflammatory drugs have been shown to be neuroprotective or anti-epileptogenic following status epilepticus. For example, blockade of leukocyte–endothelial interactions following status epilepticus (SE) via administration of α4 integrin specific antibodies reduces the occurrence of spontaneous seizures in the chronic epileptic phase (Fabene et al., 2008). Non-steroidal anti-inflammatory drugs (NSAIDs) given after SE have wide-ranging effects depending on the animal model used and schedule of administration. Parecoxib, a selective cyclooxygenase-2 (COX-2) inhibitor administered for 18 days following lithium-pilocarpine induced SE is neuroprotective (but not anti-epileptogenic) (Polascheck et al., 2010), and Celecoxib reduces neuronal injury and microglia activation when administered one day after lithium-pilocarpine induced status epilepticus (Jung et al., 2006). Conditional ablation of COX-2 in forebrain neurons leads to reduced hippocampal injury at 4 days post pilocarpine induced status epilepticus. However there is also some evidence to suggest that COX-2 is neuroprotective at 24 h following the insult (Serrano et al., 2011). SC58236, another selective COX-2 inhibitor, has no effect on cell death or microglia activation in the hippocampus when administered following electrically induced SE (Holtman et al., 2009). Therefore, there is conflicting evidence on whether modulation of inflammatory cascades can influence brain injury following status epilepticus in rats. In order for these findings to be translated into a clinical setting, there needs to be a biomarker for therapy monitoring. T_2_-weighted magnetic resonance imaging (MRI) can be used as a biomarker of vasogenic edema (Batchelor et al., 2007) and has been observed to be elevated within 2 days of childhood status epilepticus (Scott et al., 2002). In this study we investigate whether using a broad-spectrum anti-inflammatory agent (dexamethasone) can reduce vasogenic edema, assessed by quantitative transverse magnetization relaxation time constant (T_2_) measurements following pilocarpine induced SE in rats, and whether these changes predict final hippocampal volumes.

Corticosteroids such as dexamethasone (DEX) act on the glucocorticoid receptor and are highly effective in reducing BBB permeability. DEX does not readily cross the BBB (Meijer et al., 1998) and the mechanisms by which DEX is BBB protective are still not well understood and are likely to be numerous. However one mechanism by which this effect may occur could be the inhibition of NF-kappa-B activity via induction of inhibitor of kappa-B (IκB) proteins (Auphan et al., 1995). DEX appears to reduce infarct volume when administered following cerebral ischemia in rats (Bertorelli et al., 1998), supporting the view that DEX may also alleviate injury following status epilepticus. Two recent studies have found that dexamethasone is neuroprotective when administered prior to pilocarpine or lithium-pilocarpine induced status epilepticus (Al-Shorbagy et al., 2012, Marchi et al., 2011), the mechanisms of which are thought to occur via the alleviation of vasogenic edema and subsequently less severe status epilepticus. However, the clinical relevance of these studies is debatable as administration of dexamethasone preceded the insult. In the current study we investigate whether DEX is protective when administered following SE. It was found that dexamethasone led to increased T_2_ at 2 days and 4 days following SE compared to controls and to a subsequent worsening of brain injury.

## Materials and methods

### Animal model

All animal procedures were carried out in accordance with the UK Animals (Scientific Procedures) 1986 Act and institutional ethics regulations.

#### Experiment 1

Unless otherwise stated, all chemicals were obtained from Sigma-Aldrich, UK. Male Sprague–Dawley rats (170–210 g) were obtained from Charles River Laboratories (Margate, UK) (n = 42) and were kept under controlled environmental conditions including a 12 h light–dark cycle and the provision of with food and water ad libitum. Lithium chloride (3 meq/kg, intraperitoneal (i.p.)) was administered approximately 3 h prior to pilocarpine. Rats were then pre-treated with methyl scopolamine nitrate (5 mg/kg, i.p.) in order to reduce the peripheral effects of pilocarpine. 30 min later, pilocarpine hydrochloride (30 mg/kg, i.p.) was given in order to induce status epilepticus. Saline was administered in control animals in place of pilocarpine (n = 4). Seizure severity was assessed every 10 min using the Racine scale (Racine, 1972). Status epilepticus was defined as stage 3 on the Racine scale. Diazepam (10 mg/kg, i.p., Hameln Pharmaceuticals, Gloucester) was administered 90 min following SE onset in order to terminate the seizure. Additional diazepam (10 mg/kg) was administered 30–40 min later. Following status epilepticus, rats were randomly assigned to one of two groups: SE-DEX10 rats (n = 13) received dexamethasone sodium phosphate (10 mg/kg, equivalent to 7.6 mg/kg dexamethasone) immediately following status epilepticus and at 24 h (10 mg/kg) following SE. This dose is comparable to doses that have shown or attempted to demonstrate efficacy in other rat models of brain edema (Altman et al., 1984, Bertorelli et al., 1998, Mima and Shigeno, 2000, Shapira et al., 1988, Tran et al., 2010). SE rats (n = 15) received saline injections in place of dexamethasone. Dexamethasone is known to have a diuretic effect (Liu et al., 2010), therefore SE-DEX10 and SE animals received subcutaneous saline and saline/glucose solution for the first few days following SE.

#### Experiment 2

As a high mortality rate was observed in the SE-DEX10 group, the experiment was repeated using the same protocol except a single dose of dexamethasone sodium phosphate (2 mg/kg) was administered i.p. at 1 h following SE (n = 16). A single dose was used in Experiment 2 in order to minimise exposure to possible side effects or the stress caused by such side effects. Following SE rats were randomly assigned to one of two groups: SE (n = 6) and SE-DEX2 (n = 6).

### Magnetic resonance imaging

MRI relaxometry was performed at 48 h and 96 h following SE. A subset of animals in Experiment 1 were imaged prior to SE induction (n = 17) and all of these rats were used in the data analysis. High resolution structural imaging was conducted between 18 and 20 days following SE but was not performed at the earlier time points in order to keep the imaging protocol and exposure to isoflurane short. The extremely short (5 min) protocol enabled rats to be imaged as close as possible to the 48 h and 96 h time points. All imaging was achieved using a 9.4 Tesla DirectDrive VNMRS horizontal bore scanner with shielded gradient system (Agilent Technologies, Palo Alto, CA) and a 4-channel rat head phased-array coil (Rapid Biomedical GmbH, Würzburg, Germany). Animals were anesthetised with 4% isoflurane and maintained at 1.5–2% isoflurane in pure oxygen (1 L/min) throughout the imaging protocol. A physiological monitoring system (SA Instruments, Stony Brook, NY) was used to monitor respiration rate and rectal temperature. Temperature was maintained at 37 ± 0.5 °C using an air and water tubing warming system. T_2_ measurements were performed across 15 contiguous slices using a multi-slice multi-echo spin-echo sequence using the following parameters: repetition time (TR) = 2.5 s, field of view (FOV) = 25 × 25 mm, slice thickness = 1 mm, matrix = 128 × 128 and echo time (TE) = 8, 16, 24, 32, 40, 48, 56, 64, 72, 80, 88, 96, 104, 112, 120 ms. T_2_-weighted high resolution structural imaging was performed using a 3-dimensional fast spin-echo (fse) sequence with 150 μm isotropic resolution (TR = 1.8 s, FOV = 24 × 24 × 24 mm, matrix = 160 × 160 × 160, effective echo time (TEeff) = 41.8 ms, echo-train length (etl) = 16, acquisition time = 48 min).

## Quantitative T_2_

Regions of interest were identified by coregistration of the multi-echo images to a rat brain MRI template (Schwarz et al., 2006). This was performed in SPM 8 (UCL Wellcome Trust Centre for Neuroimaging, www.fil.ion.ac.uk) using a 12 parameter affine registration with normalized mutual information as the cost function. Following coregistration, the transformation matrix was used to transform the regions of interest (ROIs) to the image space. ROIs included right and left somatosensory cortices, anterior dorsal hippocampus, caudate putamen, cingulate cortices, piriform cortices and the thalamus. Quantitative T_2_ measurements were performed by calculating power images (Miller and Joseph, 1993). For each region, the mean value from each echo time was used to fit a single exponential decay using non-linear least squares regression in MATLAB (Mathworks, Natick, MA). Odd echoes were omitted from the analysis in order to reduce errors caused by imperfect refocusing of magnetisation. All datasets fitted well to mono-exponential decay functions. An example of the fitted T_2_ data from an ROI in the cerebral cortex is shown in Appendix A.

## Volumetric measurements

The data was firstly zero filled to 256 × 256 × 256 and then Tukey filtered in k-space to reduce Gibbs ringing artefacts. Bias-field correction was then performed using N4ITK (Nick's N3 ITK Implementation For MRI Bias Field Correction) (Tustison et al., 2010). Following this, intensity normalization was conducted using the method developed by Nyul et al. (2000). Whole brain and hippocampal volume measurements were both performed automatically using a multi-atlas approach. Whole-brain volume measurements were carried out using 6 previously acquired and manually masked 3-dimensional fse datasets as atlases. These were coregistered to the target images using a block-matching affine registration algorithm (Modat et al., 2010, Ourselin et al., 2001, Ourselin et al., 2002). Label fusion was achieved by majority voting, which involves assigning each voxel the label that the majority of the candidate segmentations agree on (Heckemann et al., 2006). Hippocampal volume measurements were performed using a multi-atlas approach using first affine registration followed by non-rigid registration based on free-form deformation using B-splines. This was implemented in the NiftyReg software package using the masks generated for the whole brain measurements (Modat et al., 2010, Rueckert et al., 1999). Label fusion was conducted using the STEPS algorithm with parameters optimised on a training dataset using leave-one-out cross validation (Cardoso et al., 2013, Ma et al., 2012). The training dataset consisted of 8 previously acquired post-SE datasets and 17 control datasets. The optimum parameters for segmentation of the post-SE datasets were k = 4, where k is the width of the Gaussian kernel used as the similarity measure (local normalized cross-correlation) for atlas ranking and n = 7, where n is the number of top ranked atlases used for label fusion. These parameters resulted in an average Dice score (compared to manual segmentation) of 0.894 ± 0.0021 for controls and 0.883 ± 0.0060 for post-SE subjects. The same parameters were used for automated segmentation of the hippocampus across all 3-dimensional fse datasets. The Dice score is a similarity measure that is used here to quantify the accuracy of the automated segmentation. A score of 0.883 demonstrates that highly accurate segmentation can be achieved even in the case of significant hippocampal atrophy. Relative hippocampal volume (rHCV) was calculated by dividing the hippocampal volume by the total brain volume.

### Statistical analysis

Unless otherwise stated, statistical analysis between groups was performed using one-way ANOVA and unpaired t-tests with unequal variance in the R software package (http://www.r-project.org, version 3.1-109.). Mortality between groups was tested using the test of equal proportions in R. In order to account for correlation between variables, T_2_ was compared across all regions by means of a linear-mixed effects model using the lme4 (mixed-effects modelling with R) package in the R environment (Pinheiro et al., 2013). For all linear-mixed effects models, rats were considered to be random effects, whereas treatment groups, weights, study date and mortality were considered to be possible fixed effects (i.e. explanatory variables). In a stepwise mixed effects model framework, an initial model starting with T_2_ across all brain regions is used to identify significant regions as the explanatory variables for hippocampal volume. Box and whisker plots show the median, and first and third quartiles. The whiskers are drawn from the first and third quartiles to the most extreme point not considered outliers. Outliers are considered to be more than 1.5 interquartile ranges from either the first or third quartile and are marked with a plus sign. Averages are expressed as the mean ± S.E.M. Statistical significance was assigned at p < 0.05.

## Results

The aims of Experiment 1 were to test the effects of dexamethasone given following SE at a dose of 10 mg/kg on T_2_ and hippocampal volume. Surprisingly, the results from Experiment 1 suggested that dexamethasone at 10 mg/kg had a detrimental effect on both survival during the first week after SE and brain injury at 3 weeks. For this reason, Experiment 1 was repeated using a lower dose of 2 mg/kg DEX.

### Animal model

**Fig. 1:**
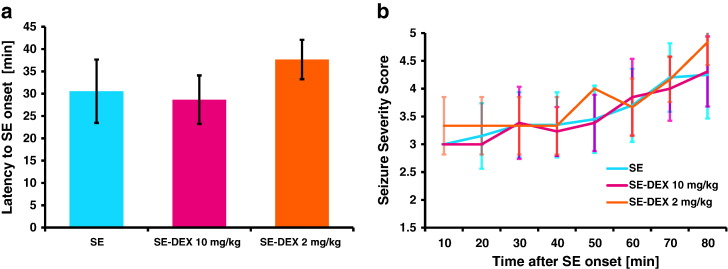
Behavioral assessment of status epilepticus in SE and SE-DEX rats. (a): Latency to onset of status epilepticus. (b): Behavioral assessment of seizure severity throughout status epilepticus. (a) and (b): SE (n = 21), SE-DEX 10 mg/kg (n = 13), SE-DEX 2 mg/kg (n = 6). Data are displayed as mean ± standard deviation.

All of the rats that were administered pilocarpine progressed to status epilepticus. None of the animals in the control group exhibited any behavioral change. 14 out of 54 (26%) animals died during or immediately after SE and were therefore excluded from the study. There was no difference between the three groups: SE, SE-DEX2 and SE-DEX10 in regards to latency to SE onset (Fig. 1a). Furthermore, there was no difference in seizure severity according to the behavioral scoring (Fig. 1b). All rats were scored throughout each 10 minute time period. During the later stages of status epilepticus almost all rats reached stage 5 on the Racine scale, during which time seizure severity alternated between stages 3 and 5. Behavioral scoring after 80 min was 4.3, 4.8 and 4.3 for SE, SE-DEX2 and SE-DEX10 respectively. The two doses of diazepam were effective at terminating SE in all rats and no overt signs of sustained seizures could be observed within the following 24 h. Dexamethasone or saline was administered at 1 h following diazepam injection. At this time point, rats were lethargic but there were no signs of on-going seizure-like activity.

### Delayed mortality

Within the first week after SE, 3 of 21 subjects died in the SE group, 2 of 6 in the SE-DEX2 group and 6 of 13 in the SE-DEX10 group. The proportion of rats that died within the week following status epilepticus was therefore 0.14, 0.33 and 0.46 for SE, SE-DEX2 and SE-DEX10 respectively. The animals that died after the 2 and 4 day imaging time points were included in the analysis. Although these proportions were not significantly different (p = 0.11), these data indicate that dexamethasone may exacerbate mortality associated with status epilepticus.

### Time course of T_2_ alterations

**Fig. 2:**
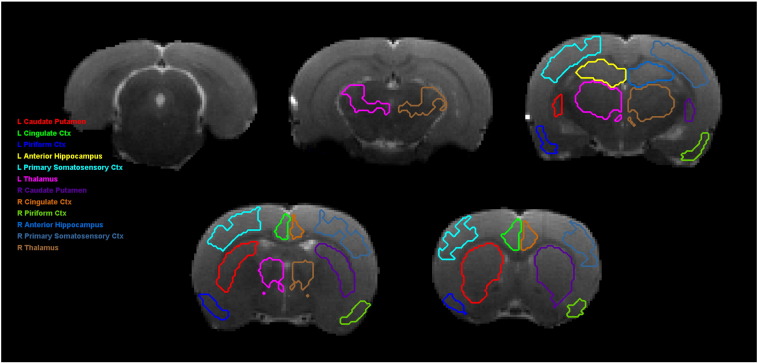
Automatic segmentation of MRI images used for quantitative T_2_ measurements. Automated segmentation was performed via affine coregistration to a rat brain template. Regions were transformed to the image space and are shown here on coronal MRI images with a slice thickness of 1 mm. For display purposes, the image shown is the image averaged across all echo times in the multi-echo sequence. Images are shown for alternate slices.

**Fig. 3:**
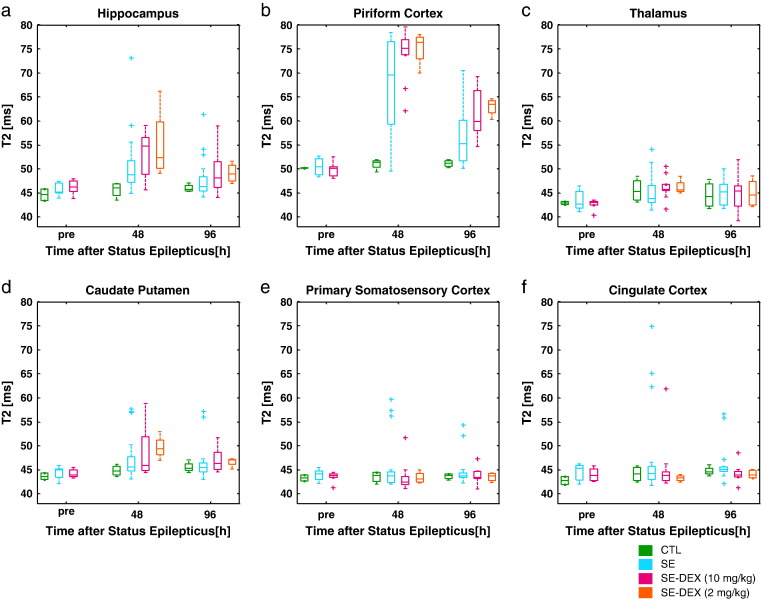
T_2_ relaxation times measured pre, 48 h and 96 h following lithium-pilocarpine induced status epilepticus. (a) hippocampus, (b) piriform cortex, (c) thalamus, (d) caudate putamen, (e) primary somatosensory cortex, (f) cingulate cortex. Treatment groups: CTL (n = 4), SE (n = 20), SE-DEX10 (n = 10), SE-DEX2 (n = 4).

**Fig. 4:**
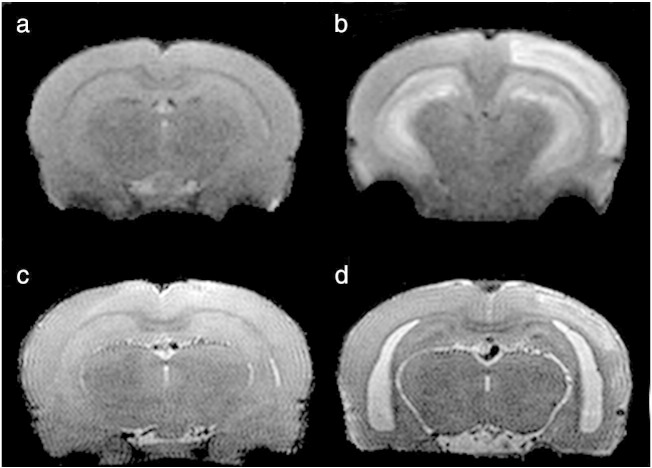
Coronal MRI images of the rat brain following status epilepticus demonstrating early edema and later hippocampal injury. First column: Control. Second column: post status epilepticus. (a) and (b): low resolution images acquired at 48 h following administration of saline or pilocarpine respectively. (c) and (d): high resolution images acquired 3 weeks after saline or pilocarpine respectively. (b) Illustrates marked bilateral edema in the hippocampus and unilateral edema in the neocortex. (d) shows significant atrophy of the hippocampus leading to enlargement of the lateral ventricles. Hypointense regions in the CA1,CA3 and dentate gyrus of the hippocampus are evident and hyperintense regions in the right neocortex correspond to the regions of hyperintensity that occurred at 2 days following SE.

Automated segmentation of the rat brain was achieved by rigid coregistration to a rat brain template. The accuracy of the segmentation was verified visually (Fig. 2). T_2_ is elevated at 48 h in SE rats compared to CTL in the hippocampus (52.1 ms vs. 45.6 ms, p = 0.008) (Fig. 3a) and piriform cortex (69.5 ms vs. 51.0 ms, p = 5 × 10^− 7^) (Fig. 3b). These alterations began to normalize by 96 h. This marked bilateral hippocampal edema is shown in Fig. 4b. Four animals in this study exhibited marked unilateral edema in the neocortex (Fig. 4b) that was not present in controls (Fig. 4a). These can be seen as outliers in Figs. 3e and f. MRI performed at 3 weeks following the initial insult (Fig. 4d) exhibits significant atrophy of the hippocampus leading to enlargement of the lateral ventricles. Hypointense regions in the CA1, CA3 and dentate gyrus of the hippocampus are evident (Fig. 4d). Hyperintense regions at the three week time point in the right neocortex (Fig. 4d) correspond to the regions of hyperintensity that occurred at 48 h following SE (Fig. 4b).

### Effect of dexamethasone on acute cerebral edema

One of the aims of this study was to test whether dexamethasone alleviates the regional T_2_ caused by status epilepticus in the hippocampus and piriform cortex. The mean T_2_ relaxation time at 2 days in the hippocampus was: 45.6 ms, 52.1 ms, 55.3 ms, 55.0 ms for CTL, SE, SE-DEX10 and SE-DEX2 respectively (Fig. 3a). In the piriform cortex, T_2_ at 2 days was: 51.0 ms, 69.5 ms, 74.3 ms and 75.2 ms for CTL, SE, SE-DEX10 and SE-DEX2 respectively (Fig. 3b). The SE-DEX10 group had significantly higher T_2_ in the piriform cortex than SE at 2 days and 4 days (p = 0.01, p = 0.04 respectively).

A linear mixed-effects model was also used to compare all 12 brain regions together in the treatment groups to the CTL group in order to account for other possible within-sample effects and repeated measures. SE-DEX10 was significantly different to CTL at day 2 and day 4 (p = 0.004 and 0.03), DEX2 was significantly different to CTL on day 2 (p = 0.01) but not day 4 and SE was not significantly different from the CTL group at either time point.

### Effect of dexamethasone on relative hippocampal volume and total brain volume

**Fig. 5:**
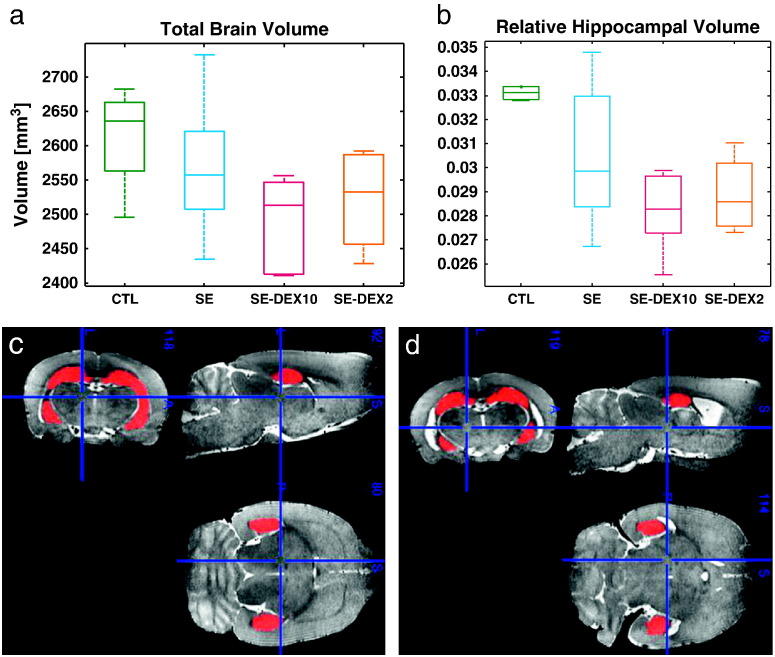
Total brain volume and relative hippocampal volume at 3 weeks after status epilepticus. (a) Total brain volume *vs*. treatment group. (b) Relative hippocampal volume *vs*. treatment group. (c) Example image showing automated segmentation of the hippocampus in a control rat. (d) Example image demonstrating automated segmentation of the hippocampus in a post-status epilepticus rat, 3 weeks after the initial insult. Treatment groups: CTL (n = 4), SE (n = 16), SE-DEX10 (n = 5), SE-DEX2 (n = 4).

Accurate segmentation of the hippocampus was achieved in MRI images of both controls and post-status epilepticus rats (Figs. 5c and d) using a multi-atlas approach. At 3 weeks following status epilepticus, rHCV was significantly different among groups (Fig. 5b, p = 0.01). SE rats had significantly lower relative hippocampal volume compared to CTL (p = 0.001). SE-DEX10 had significantly lower rHCV compared to SE (p = 0.003). There was no significant difference between SE-DEX2 and SE. Total brain volumes were 2612.8 ± 40.4 mm^3^, 2565 ± 12.3 mm^3^, 2487.7 ± 11.7 mm^3^ and 2521.9 ± 12.18 mm^3^ for CTL, SE, SE-DEX10 and SE-DEX2 respectively (Fig. 5a). DEX appeared to be detrimental to total brain volume, however brain volumes across all groups were not significantly different (p = 0.09). There was no significant difference in weight change over three weeks between any of the groups (p = 0.25).

### Relationship between T_2_ and hippocampal volume

**Fig. 6:**
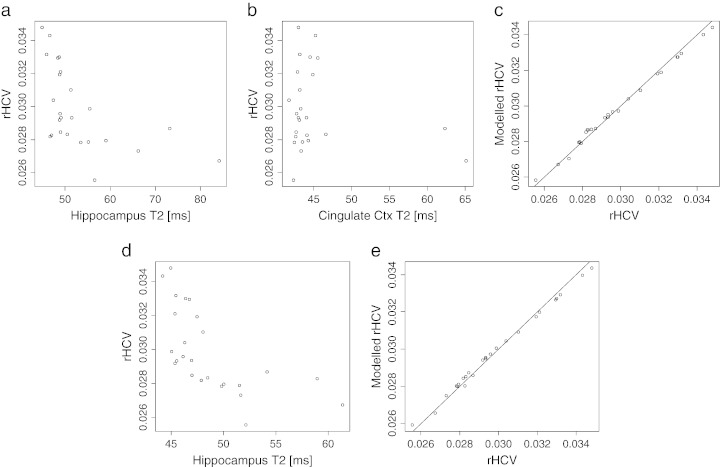
Scatter plots showing the relationship between early T_2_ measurements and relative hippocampal volume (rHCV) measured 3 weeks after SE. (a) and (b): Hippocampus and cingulate ctx T_2_ measured 2 days after SE *vs*. rHCV. (c) rHCV *vs*. rHCV modelled using a linear mixed-effects model for the data shown in (a) and (b). (d) Hippocampus T_2_ measured 4 days after SE *vs*. rHCV. (e) rHCV *vs*. rHCV modelled using a linear mixed-effects model for the data shown in (d).

According to the linear mixed-effects model and considering all animals that underwent status epilepticus, T_2_ in the hippocampus and the cingulate cortex at 2 days following status epilepticus is predictive of relative hippocampal volume at 3 weeks (Fig. 6a) (β_1_ = − 0.00030 ± 0.00007, 0.00031 ± 0.0001, p = 0.0004 and p = 0.01 respectively). T_2_ at 4 days following status epilepticus in the hippocampus is also predictive of relative hippocampal volume (Fig. 6b) (β_1_ = − 0.00035 ± 0.00009, p = 0.0007).

## Discussion

The main findings from this study are that administering dexamethasone following lithium-pilocarpine induced status epilepticus regionally intensifies acute T_2_ alterations at 2 days and 4 days following status epilepticus. Second, dexamethasone adversely affects the degree of hippocampal injury at 3 weeks following SE. Third, dexamethasone treated rats show a trend toward increasing mortality associated with status epilepticus. These findings apply even at doses of dexamethasone sodium phosphate as low as 2 mg/kg. Importantly, T_2_ relaxation times in the hippocampus were found to be predictive of later hippocampal injury. Lastly, this study demonstrates that quantitative T_2_ measurements and volumetric measurements in the rat can be performed using automated methods that require no user intervention.

Previous studies have shown the T_2_ alterations following status epilepticus in the rat peak at around 2 days following SE (Choy et al., 2010, Roch et al., 2002), and this is confirmed in the current study. Our previous work demonstrates that although there are significant changes in T_2_ in the piriform cortices at 1 day following SE (Duffy et al., 2012), T_2_ alterations in the hippocampus appear to not be evident until 2 days following SE. The current study supports a previous study revealing that T_2_ in the hippocampus at 2 days is predictive of later hippocampal volume (Choy et al., 2010).

This study has demonstrated that the acute T_2_ alterations that occur following lithium-pilocarpine induced status epilepticus are exacerbated by DEX in the regions already affected by SE. Although other studies have looked at the effects of pre-treatment with DEX on SE severity (Marchi et al., 2011), to our knowledge this is the first study to test whether DEX is protective following SE. The design of the current study did not enable us to determine whether these effects are due to side-effects of the drug such as dehydration, alterations in plasma osmolality or blood pressure effects. No relationship was found between treatment groups and the differences in body weight between pre-SE and any of the subsequent days, suggesting that dehydration was not the cause of the observed outcomes. It is also worth noting that the diuretic effect of DEX in conjunction with its anti-inflammatory effect should help to alleviate vasogenic edema (Liu et al., 2010).

Unpredictably, a trend toward increasing delayed mortality rate was observed in rats treated with dexamethasone. The mechanisms behind delayed mortality following status epilepticus are unknown. It has been speculated that it could be caused by recurrent seizures (Levin et al., 2012). Vasogenic edema is well-known to contribute to death in neurological disorders, therefore the trend toward increased mortality in DEX treated rats compared to the SE group could be attributable to increased brain edema.

Several possible explanations exist for the results observed. First, these data could suggest that some early inflammatory processes may provide a protective effect, which is not inconceivable given that there is an extremely fine balance between the beneficial and detrimental effects of neuroinflammation (Rolls et al., 2009, Serrano et al., 2011). Also notable is that pre-treatment with COX-2 inhibitors has been observed to lead to increased mortality both in electrically induced status epilepticus and in the kainate model (Baik et al., 1999, Holtman et al., 2010). This effect was likely due to aggravation of seizure activity, which is unlikely to be the case in the current study given that previous data indicates that dexamethasone suppresses pilocarpine induced SE (Marchi et al., 2011). Further supporting the hypothesis that early inflammation is protective, is a study which showed that Interleukin-6 knockout mice exhibit reduced astrogliosis compared to wildtypes following kainate induced seizures. This was accompanied by dramatically intensified neuronal degeneration in the hippocampus (Penkowa et al., 2001). It is also noteworthy that randomised controlled trials of anti-inflammatory therapies in patients with stroke or traumatic brain injury (TBI) have observed an increase in mortality rate in patients administered anti-inflammatory drugs compared to those given a placebo (Edwards et al., 2005, Sherman et al., 2001). One possible explanation for the worse outcome in TBI patients treated with corticosteroids is that steroids have recently been shown to worsen cerebral edema under acidotic conditions in the rat (Tran et al., 2010). Further research is required to explore these hypotheses.

An alternative explanation is that DEX could elicit a pro-inflammatory response. It has been shown that glucocorticoids can exhibit pro-inflammatory effects under certain conditions, e.g. chronic stress (Munhoz et al., 2006). Furthermore, corticosterone treatment has been shown to exacerbate kainic acid induced neurotoxicity in the CA3 region of the hippocampus and concomitantly increase the number of immunoreactive cells (Dinkel et al., 2003). Given that the current study also reports a cerebral insult that is excitotoxic in nature, possible pro-inflammatory effects of DEX might contribute to exacerbated brain injury in DEX treated rats compared to saline treated rats.

Finally this study has demonstrated that automated methods of data analysis are effective tools that can be employed in epilepsy research to reduce the laboriousness of manual region drawing and therefore can be used in conjunction with histopathology to test the neuroprotective efficacy of new experimental treatments. This is a noteworthy development as although other studies have applied nonlinear registration (Gaser et al., 2012), to our knowledge all other studies that have attempted to measure hippocampal volumes in models of epilepsy have employed manual segmentation.

## Conclusions

In conclusion, we have shown that even low doses of dexamethasone worsen the regional effects that status epilepticus has on the brain. This may be a forewarning that if anti-inflammatory drugs such as corticosteroids are to be used in clinic during or following childhood status epilepticus as has been advocated (Ravizza et al., 2011), the type of drug and time course of administration are likely to be crucial.

The following are the supplementary data related to this article.

Appendix A Effect on T_2_ by fitting points from 3 to 7 echo times.
T_2_ = 42.22, 43.18, 43.24, 43.29 and 43.43 ms for (a)–(e) respectively.
