# Supplementary figures and images for: Dexamethasone exacerbates cerebral edema and brain injury following lithium-pilocarpine induced status epilepticus
Source: Neurobiol Dis. 2014 Mar;63:229–36. doi: 10.1016/j.nbd.2013.12.001 (PMC3905166; doi:10.1016/j.nbd.2013.12.001)

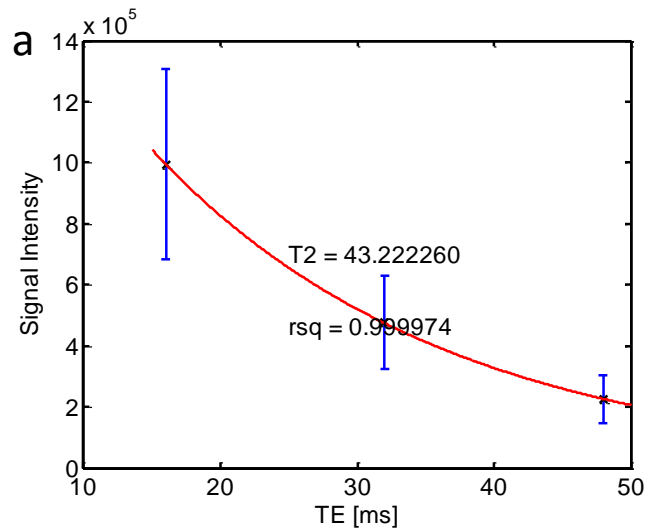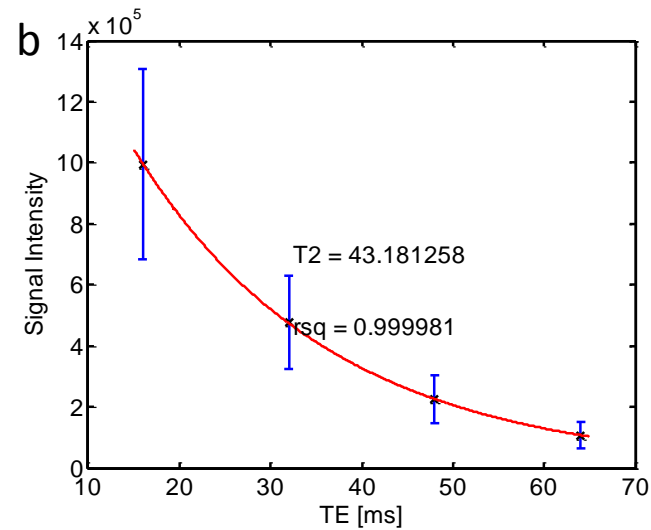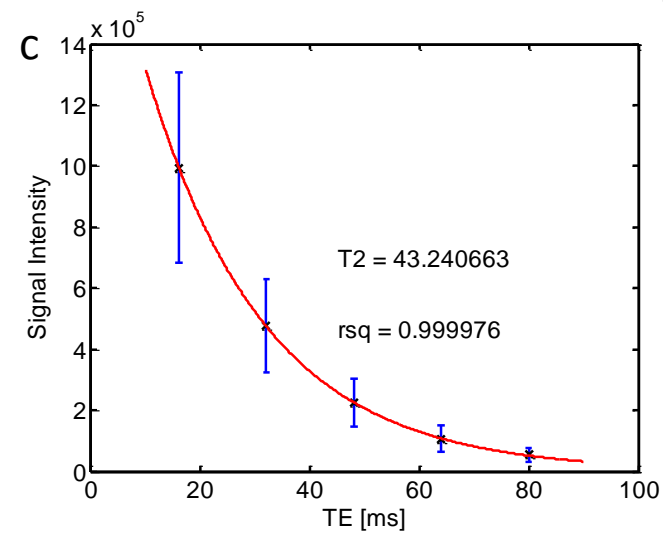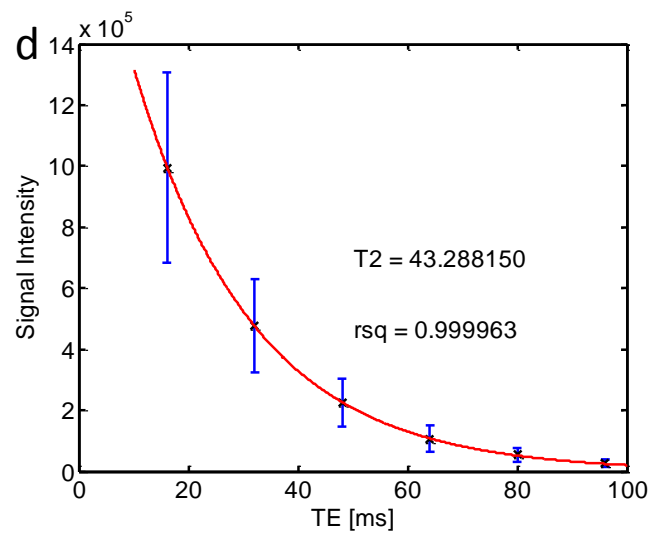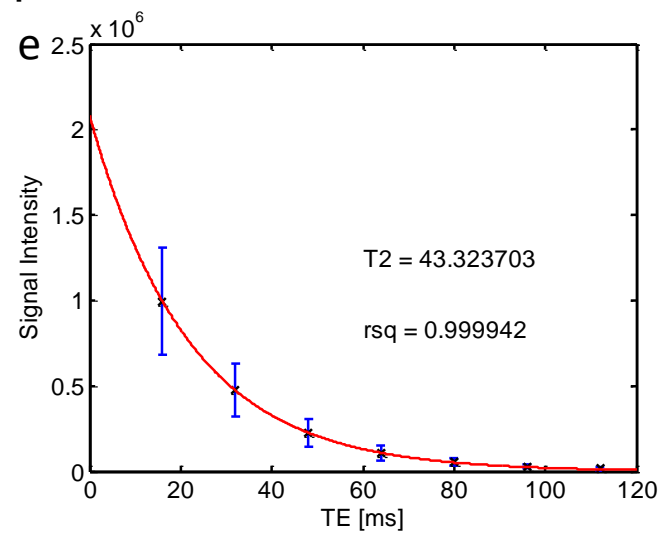

Supplement: Appendix A — Effect on T2 by fitting points from 3 to 7 echo times. T2 = 42.22, 43.18, 43.24, 43.29 and 43.43 ms for (a)–(e) respectively. [file mmc1.pdf]
